# Comparison of a Daily Smartphone App and Retrospective Questionnaire Measures of Adherence to Nicotine Replacement Therapy Among Pregnant Women: Observational Study

**DOI:** 10.2196/35045

**Published:** 2023-03-07

**Authors:** Joanne Emery, Yue Huang, Felix Naughton, Sue Cooper, Lisa McDaid, Anne Dickinson, Miranda Clark, Darren Kinahan-Goodwin, Ross Thomson, Lucy Phillips, Sarah Lewis, Tim Coleman

**Affiliations:** 1 School of Health Sciences University of East Anglia Norwich United Kingdom; 2 Centre for Academic Primary Care University of Nottingham Nottingham United Kingdom; 3 Adult Social Care and Health Derbyshire County Council Matlock United Kingdom; 4 Division of Epidemiology and Public Health University of Nottingham Nottingham United Kingdom

**Keywords:** smoking cessation, pregnancy, nicotine replacement therapy, treatment adherence measurement, smartphone app, questionnaires, ecological momentary assessment, mHealth, mobile health, smoking, nicotine

## Abstract

**Background:**

Few studies have investigated how to best measure adherence to smoking cessation medications, but continuous usage measures are recommended.

**Objective:**

In this first study of its kind, we compared methods for measuring adherence to nicotine replacement therapy (NRT) among pregnant women, investigating the completeness and validity of data collected from daily assessments using a smartphone app versus data collected from retrospective questionnaires.

**Methods:**

Women aged ≥16 years who were daily smokers and <25 weeks pregnant were offered smoking-cessation counseling and encouraged to use NRT. For 28 days after setting a quit date (QD), women were asked to report NRT use daily to a smartphone app and to questionnaires administered in person or remotely at 7 and 28 days. For both data collection methods, we provided up to £25 (~US $30) as compensation for the time taken providing research data. Data completeness and NRT use reported to the app and in questionnaires were compared. For each method, we also correlated mean daily nicotine doses reported within 7 days of the QD with Day 7 saliva cotinine concentrations.

**Results:**

Of the 438 women assessed for eligibility, 40 participated and 35 accepted NRT. More participants (31/35) submitted NRT usage data to the app by Day 28 (median 25, IQR 11 days) than completed the Day 28 questionnaire (24/35) or either of the two questionnaires (27/35). Data submitted to the app showed a lower reported duration of NRT use compared to that indicated in the questionnaire (median for app 24 days, IQR 10.25; median for questionnaire 28 days, IQR 4.75; *P*=.007), and there appeared to be specific cases of overreporting to the questionnaire. Mean daily nicotine doses between the QD and Day 7 were lower when calculated using app data (median for app 40 mg, IQR 52.1; median for questionnaire 40 mg, IQR 63.1; *P*=.001), and some large outliers were evident for the questionnaire. Mean daily nicotine doses, adjusted for cigarettes smoked, were not associated with cotinine concentrations for either method (app *r_s_*=0.184, *P*=.55; questionnaire *r_s_*=0.031, *P*=.92), but the small sample size meant that the analysis was likely underpowered.

**Conclusions:**

Daily assessment of NRT use via a smartphone app facilitated more complete data (a higher response rate) than questionnaires, and reporting rates over 28 days were encouraging among pregnant women. App data had better face validity; retrospective questionnaires appeared to overestimate NRT use for some participants.

## Introduction

Smoking in pregnancy is an avoidable cause of miscarriage; stillbirth; prematurity; low birth weight; perinatal, neonatal, and sudden infant death; and poorer infant cognition and behavioral outcomes [1-3]. Effective cessation interventions reduce low birth weight and special care admissions [4]. Smoking in pregnancy is a major international public health problem; the prevalence is 13% to 25% in high-income countries [5-9] and a similar epidemic is developing in low- and middle-income countries [10]. Stopping smoking prevents harms, and nicotine replacement therapy (NRT) is widely used for cessation in pregnancy because it is very plausibly less harmful than smoking and can help women to quit [11]. However, reviews suggest that NRT is less effective in pregnancy than in the general population [11,12], most likely due to insufficient dosing. Nicotine metabolism accelerates in pregnancy, peaking by 18 weeks gestation and falling to prepregnancy rates after childbirth [13,14]. In addition, compared to smoking, NRT provides pregnant women with a much lower nicotine dose (70.3 ng/mL lower mean cotinine concentration) [15]. Hence, pregnant women quitting smoking with NRT likely need higher doses to ameliorate nicotine withdrawal, without which they may discontinue NRT, perceiving it as ineffective and making relapse to smoking more likely.

Adherence to NRT in sufficiently high doses appears to be important for achieving smoking abstinence [16]; however, NRT adherence observed among pregnant women is poor. In trials enrolling pregnant smokers, only 7% to 30% finished NRT courses [17]. Adherence to NRT in pregnancy within routine care is also poor; although UK general practitioners can supply NRT in courses that are sufficiently long to be effective, over two-thirds of UK pregnant women seek only a single prescription for a 2-week supply, which is likely too short to help them [18]. In contrast, nonpregnant trial participants have exhibited up to 94% adherence with studies’ NRT regimens [19]. A Cochrane review of nonpregnant smokers found that interventions for increasing adherence with smoking cessation therapies might increase smoking cessation [20]; thus, better adherence to NRT in pregnancy is likely to improve quit attempt success. Greater nicotine dependence and higher rates of nicotine metabolism are inversely associated with cessation in pregnancy [21,22], and the negative impacts of both factors could be minimized by using NRT for long enough at sufficiently high doses.

Measurement of NRT adherence in pregnancy is important for researchers planning interventions to increase adherence. However, the measurement of adherence to NRT is a nascent science. The Cochrane review recommended continuous measures of drug use duration as adherence outcomes [20]. As the duration of NRT use is associated with future cessation, such measures could be appropriate for monitoring NRT adherence. For example, “days of use of NRT” by pregnant [23] and nonpregnant quitters [24] is strongly associated with stopping smoking and this association is considered causal among nonpregnant quitters [16,19]. The dose (intensity) of NRT used early in quit attempts is also associated with cessation [25,26], and is another aspect of NRT use that could be appropriate as an adherence outcome. However, there are currently no gold-standard NRT adherence outcome measures, and there has been little attention given to the best methods of seeking and recording continuous adherence data [20]. Some studies have required participants to recall drug use over long periods at follow-up [20]. However, for self-report measures, questions that occur close in time to the behavior (“momentary” questions) are typically considered more accurate than questions with long recall periods [27]. This is a crux of the ecological momentary assessment (EMA) [27] approach, which seeks repeated assessments of a behavior close in time to its occurrence. 

Using data from a study carried out in a routine clinical setting in which pregnant women reported the duration and intensity of their NRT use during a quit attempt, we investigated the completeness and validity of data obtained after regular requests from a smartphone app and less frequent requests from retrospective questionnaires.

## Methods

### Overview

We developed a behavioral intervention called “Baby, Me & NRT” (BMN), which aims to improve pregnant women’s use of, and adherence to, NRT. We collected the data analyzed in this study during three sequential cohort studies in which researchers delivered BMN to pregnant women and sought feedback for intervention optimization. Between cohorts, we made some minor changes to the data collection procedures in response to participant feedback and COVID-19 restrictions.

### Recruitment and Follow-up Data Collection

The methods are fully described in the study protocol [28]. Participants were aged 16 years or over and were less than 25 weeks pregnant, smoked at least one daily cigarette (prepregnancy ≥10 cigarettes), were not currently using an electronic cigarette (e-Cigarette), and agreed to use NRT to try to stop smoking. We aimed to recruit around 40 women across all cohorts. We identified participants as they attended Nottingham University Hospitals Trust antenatal clinics. In the final cohort, due to COVID-19 restrictions on face-to-face recruitment, participants were also identified via Facebook advertisements and remotely from referrals made to the local stop-smoking service. Study dates, recruitment, and follow-up timings per cohort are given in Table S1 of Multimedia Appendix 1. In Cohorts 1 and 2, researchers sought questionnaire data in person or by telephone, whereas in Cohort 3, these data were obtained by online surveys or telephone due to COVID-19 restrictions.

At baseline, we asked women’s age, ethnicity, education, gestation, current and prepregnancy smoking behavior, nicotine dependence [29], previous experience of using NRT, partner smoking status, and concerns and “necessity beliefs” regarding NRT use. We helped participants to install a smartphone app for reporting daily NRT use (“NicUse” app) [30]. At baseline and Day 7 after their quit date (QD), we collected saliva samples for a cotinine assay, using remote methods where necessary.

### Provision of NRT

NRT was offered for 28 days in a manner consistent with the UK standard treatment for smoking cessation in pregnancy [31]. We supplied a daily NRT patch (Nicorette 16-hour 15 mg or 25 mg; NiQuitin 24-hour 14 mg or 21 mg) plus one of the following fast-acting NRTs: Nicorette Cools Lozenges (2 mg or 4 mg), Nicorette Inhalator (15 mg), Nicorette QuickMist mouth spray (1 mg/spray; only in Cohort 3). Researchers advised women to continue using NRT during brief smoking lapses of less than 14 days and, if they preferred, to leave 24-hour patches on overnight. We recorded the type(s) and strengths of NRT accepted by each participant and any subsequent changes in products issued. We did not require participants to return unused NRT or packaging from the NRT they claimed to have used.

### NRT Adherence Data Collection

#### NicUse Smartphone App

**Figure 1:**
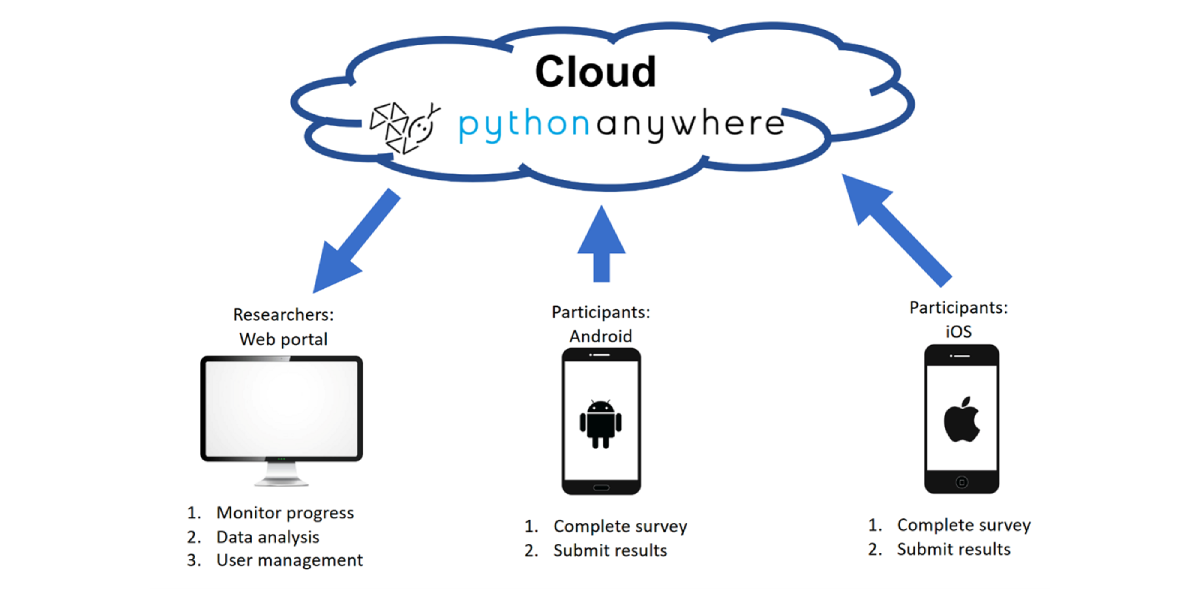
Key components of the NicUse app and directions of data transfers.

Participants downloaded the NicUse app (Figure 1) from Google Play Store (Android) or App Store (iOS). In summary, each day, we asked NicUse users to record whether or not they had used NRT, smoked, and/or vaped on the previous day. NicUse asks about the previous day’s use, from waking until going to bed, as feedback from app development work [30] suggested this was clearer than asking about specific 24-hour periods spanning 2 days (eg, 6 PM to 6 PM). This can be seen as a form of EMA [27] (ie, repeated assessment of a behavior close in time to its occurrence). Users could set a daily reminder, at a time of their choice, for NicUse to prompt them to report. Participants who reported using NRT on the previous day were asked which type(s) and how much they had used, and those who reported smoking on any day were asked how many cigarettes were smoked. The specific app items are given in Multimedia Appendix 1. Users were only permitted to submit survey responses within 48 hours of a day ending; after submission, entries were “locked” and anonymized data were sent to a cloud-hosted database and not stored on the app. Prior to submission, users could review and change their data, but after “locking” they were not permitted to see their entries.

For 28 days from their QD, irrespective of smoking or vaping behaviors or whether they used any NRT, we asked participants to provide daily NicUse data. To compensate women for time spent entering data into NicUse they were awarded £0.50 (£1≈US $1.20) “credits,” paid as shopping vouchers at the end of the study, for each submitted survey, with an additional £1.50 for submitting seven consecutive daily reports and £5 more for reporting on all requested 28 days (potential maximum compensation of £25). If any participant failed to submit a survey within a 48-hour period, up to three reminder text messages were sent at 24-hour intervals unless survey completion resumed. Where NicUse surveys were not completed on a particular day, data were recorded as “missing.”

#### Questionnaire

Questionnaire items, and a summary of how questionnaire data were collected in each cohort, are given in Multimedia Appendix 1. Items were worded as closely as possible to those on the app, but the wording used reflected different recall periods. In Cohort 1, we sought adherence data weekly for 4 weeks after the QD and asked about NRT use in the previous 7 days. In Cohorts 2 and 3, we sought data at 7 and 28 days after the QD and asked about the previous 7 and 28 days, respectively. Questionnaire responses were accepted until the study ended. In Cohort 3, we changed from asking for the “number of patches” used to the “number of days on which patches were used,” since with the former phrasing, some participants whose patches fell off and were replaced reported using two or more patches in 24 hours. In Cohort 1, researchers used telephone call reminders to nonrespondents; in later cohorts, we used text messages and email reminders, only telephoning nonrespondents if required. For time spent completing the Day 28 questionnaire and participation in an end-of-study telephone interview seeking views on the BMN intervention, participants received a £25 shopping voucher; this was additional to vouchers received for app completion.

### Ethical Considerations

All participants gave written informed consent prior to the start of the baseline study appointment. For Cohort 1 and Cohort 2, consent was given face to face. For Cohort 3, due to COVID-19 restrictions, consent was given via an online form. Ethical approval was granted by Nottingham 1 Research Ethics Committee (Integrated Research Application System number 254560; REC reference 9/EM/0193).

### Analyses

#### Design Overview

All analyses were exploratory. We investigated which data collection method provided the most *complete* information on “days of use” (ie, duration) of NRT, and the most *valid* data for measuring the duration and also the dose of NRT (ie, “intensity” of use between QD and Day 7). We planned to use nonparametric methods for comparing reported NRT use between methods due to anticipated nonnormality of the data (high and low adherers to NRT within the sample). All statistical tests were two-tailed.

#### Data Completeness

We report app and questionnaire response rates as the number of participants providing *any* NRT usage data, per method, for the Days 1 to 7 and Days 1 to 28 recall periods. For the app, we report the median number of days on which reports were submitted (out of 28) and, at the individual participant level, the number of days on which “any” or “no” NRT use was reported and the number of days on which data were missing.

#### Data Validity

For the duration of NRT use, we calculated the average number of days of NRT use reported to the app and questionnaire over participants for the Day 28 recall period; we compared these data using a nonparametric within-groups test (Wilcoxon signed-rank test). We also qualitatively inspected the values reported by individual participants, per method, for both the Day 7 and Day 28 recall periods.

For intensity of NRT use, using participants’ app and questionnaire data for the Day 7 recall period, we calculated mean daily nicotine doses from NRT, per participant and per method, as one seventh of their total nicotine dose reported from patch and fast-acting NRT between QD and Day 7. We compared app- and questionnaire-derived averages over participants using a nonparametric within-groups test (Wilcoxon signed-rank test), and inspected their relationship using a scatterplot and nonparametric (Spearman rank) correlation analysis. 

Saliva cotinine concentrations reflect nicotine exposure, from smoking or NRT, in the previous 7 days [32]. Therefore, to judge the accuracy of the two measures, we investigated any correlational relationships between mean daily nicotine doses from NRT reported to each method and Day 7 cotinine concentrations for two groups: (1) in participants who reported total abstinence from smoking (not “even a puff”) between QD and Day 7; and (2) in all participants regardless of smoking status, but with adjustment for the mean number of daily cigarettes self-reported to either the app or questionnaire, as appropriate. For the app, we calculated mean daily cigarettes as one seventh of the total number of cigarettes women reported between Days 1 and 7. The app did not quantify smoking for Cohort 1, and thus these participants were excluded from this analysis. For the Day 7 questionnaire, women were asked to self-report their own daily average (“How many cigarettes per day are you smoking currently?”). e-Cigarette use was not quantified in either method, and was therefore not adjusted for, but was expected to be low as this was a baseline exclusion criterion. Smoking and e-Cigarette items for the app and questionnaire per cohort are given in Multimedia Appendix 1. For both data sources, we assumed a maximum of one patch used per day, even if participants reported more, as patches could be replaced if they fell off. For group (1), we report nonparametric (Spearman rank) correlations, whereas for group (2), we report nonparametric partial correlations controlling for the mean number of daily cigarettes self-reported to the app or questionnaire, as appropriate, between QD and Day 7.

## Results

### Participants

In Cohort 1, 50 women were assessed for eligibility and 8 (16%) participated; in Cohort 2, 189 women were assessed and 12 (6%) participated; and in Cohort 3, 199 women were assessed and 20 (10%) participated. Most nonparticipants did not meet the study inclusion criteria, which included willingness to try quitting smoking with NRT plus counseling. After providing baseline data, five women in Cohort 3 took no further part in the study; they were not offered NRT and were not included in analyses. In total, we assessed 438 women for eligibility, 40 consented to join, 35 received counseling and were offered (and accepted) NRT, and 20 provided Day 7 saliva samples. Table 1 provides the participants’ characteristics.

**Table 1:** Baseline characteristics of cohort participants who accepted nicotine replacement therapy (N=35).

Characteristic		Value
Age (years), mean (SD)^a^		30.1 (5.3)
Gestation (weeks), mean (SD)^a^		15.6 (4.2)
**Ethnicity, n (%)^b^**		
	White	33 (94)
	Other	2 (6)
**Partner who smokes, n (%)^b^**		
	No partner	5 (14)
	Partner who smokes	21 (60)
	Partner is a non-/exsmoker	9 (26)
**Smoking status in previous pregnancies, n (%)^b^**		
	No previous pregnancies	3 (9)
	Yes	29 (83)
	No	3 (9)
**Time to first cigarette after waking, n (%)^b^**		
	Within 5 minutes	10 (29)
	6-30 minutes	18 (51)
	31-59 minutes	4 (11)
	1-2 hours	2 (6)
	More than 2 hours	1 (3)
Number of cigarettes per day now, mean (SD)		8.6 (6.4)
Baseline saliva cotinine concentration, mean (SD)^c^		159.3 (82.3)

^a^N=34.

^b^Percentages may not add up to exactly 100% due to rounding.

^c^N=32.

### Data Completeness

The response rate for providing any NRT adherence data via the app (31/35, 88.6% for both recall periods) was higher than that provided via the retrospective questionnaire (26/35, 74.3% for the Day 7 questionnaire; 24/35, 68.6% for the Day 28 questionnaire; 27/35, 77% for either questionnaire). Among all participants, including those who provided no app data (N=35), the mean percentage of all daily app surveys completed over the Day 7 recall period was 75.1% (SD 35.9) and was 65% (SD 38.6) for the Day 28 recall period. The median number of days on which app surveys were completed among all participants by Day 7 was 7 (IQR 4) and was 24 (IQR 21) by Day 28. Among participants who provided any app data (n=31), the median number of days on which app surveys were completed by Day 7 was 7 (IQR 2) and was 25 (IQR 11) by Day 28. Tables S2 and S3 in Multimedia Appendix 1 show the completeness of app and questionnaire data at the individual participant level for the Day 7 and Day 28 recall periods, respectively. Of 9 participants with no Day 7 questionnaire data, 5 provided app data on a median of 3 days (range 2-5 days). Of 11 participants with no Day 28 questionnaire data, 7 provided app data on a median of 6 days (range 3-19 days).

### Data Validity

#### Duration of NRT Use

**Figure 2:**
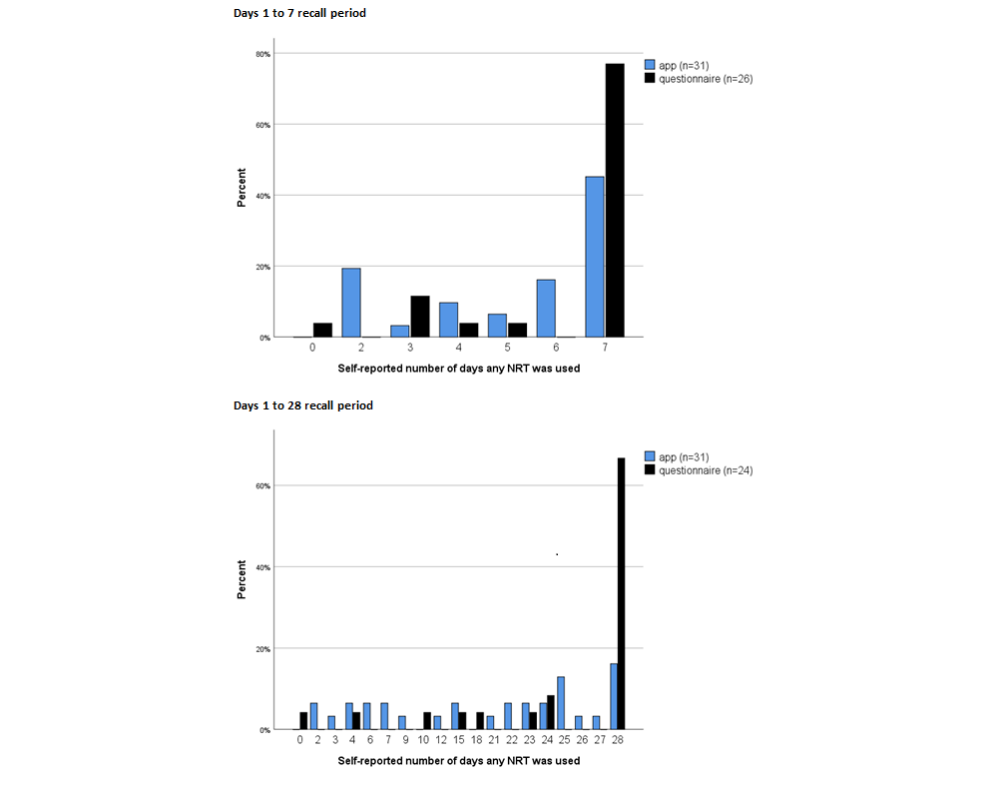
Self-reported number of days any nicotine replacement therapy (NRT) was used for Days 1 to 7 and Days 1 to 28.

Figure 2 displays distributions of the number of days (duration) of NRT use reported to the app and questionnaire for the Day 7 and Day 28 recall periods. Tables S2 and S3 in Multimedia Appendix 1 show these data at the individual participant level. Participants’ questionnaire responses are heavily skewed toward reporting that NRT was used on all 28 days. By Day 28, among those who provided both app and questionnaire data (n=24), the number of days (ie, duration) of NRT use reported by the app was significantly lower (median 24, IQR 10.25) than that reported by the questionnaire (median 28, IQR 4.75) (Wilcoxon signed-rank test *Z*=2.682, *P*<.007). Paired means were 21 (SD 7.89) for the app and 23.6 (SD 8.19) for the questionnaire. Table S2 in Multimedia Appendix 1 suggests specific cases where NRT use may have been overreported to Day 7 questionnaires; for example, participants 1203 and 1205 reported using NRT on all 7 days to questionnaires, whereas they reported not using NRT on one of these days in the app. Table S3 in Multimedia Appendix 1 suggests a greater magnitude of NRT overreporting to Day 28 questionnaires; for example, participants 1203, 1204, and 1205 reported NRT use on all 28 days to questionnaires, but reported that no NRT was used on 2, 5, and 10 days, respectively, in the app during the same period. Similarly, participant 2201 completed app surveys for 27 days and reported that no NRT was used on 23 of these days, but the corresponding questionnaire response stated that NRT was used for 10 days.

#### Intensity of NRT Use

**Figure 3:**
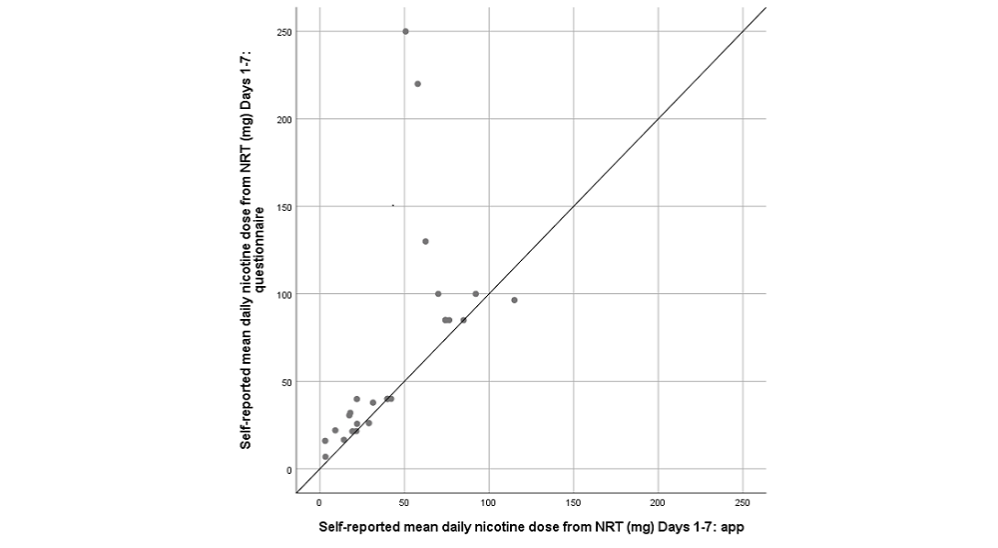
Self-reported mean daily nicotine dose (mg) from nicotine replacement therapy (NRT) reported to the app and questionnaire for Days 1 to 7 (the solid diagonal line shows the expected trend if both methods yielded equal values).

A total of 31 participants provided sufficient app data to calculate their average daily nicotine dose from NRT (in milligrams) reported between QD and Day 7; of these, 26 also provided similar data via the Day 7 questionnaire. Among those who provided both app and questionnaire data (n=26), median daily nicotine doses reported to the app and questionnaire were 40 (IQR 52.1) and 40 (IQR 63.1) mg, respectively (Wilcoxon signed rank-test *Z*=3.25, *P*=.001). Paired means were 43.5 (SD 30) and 65.1 (SD 60.2) mg, respectively. The scatterplot of app- versus questionnaire-derived nicotine doses (Figure 3) shows that, despite a strong correlation (*r_s_*=0.878, *df*=24; *P*<.001), some participants reported much higher daily NRT doses to the questionnaire than to the app, and questionnaire-derived doses appear to be higher in general.

In addition to app and questionnaire NRT dosage data, 20 participants also provided Day 7 saliva samples, and therefore cotinine concentrations: the median concentration was 109 ng/mL (IQR 106.7) and the mean concentration was 124.2 ng/mL (SD 72.1). Table 2 shows the nonparametric correlations for each data collection method between mean self-reported daily nicotine doses from NRT and Day 7 saliva cotinine concentrations among participants reporting total smoking abstinence, as well as the nonparametric partial correlations among all participants regardless of smoking status but adjusted for self-reported daily number of cigarettes. Figure S1 in Multimedia Appendix 1 displays corresponding partial regression plots. Partial correlation coefficients between self-reported nicotine doses and cotinine concentrations, adjusted for self-reported daily number of cigarettes (Table 2, “All”), were nonsignificant for both reporting methods. For participants who reported total abstinence from smoking between QD and Day 7, zero-order correlation coefficients between self-reported nicotine doses and cotinine concentrations were nonsignificant for both reporting methods, but participant numbers fulfilling this criterion were very low (Table 2).

Table 3 shows smoking status, NRT, and e-Cigarette use reported to the app or questionnaire in the first 7 days post the QD.

**Table 2:** Spearman correlations between Day 7 cotinine concentration and mean daily nicotine dose from nicotine replacement therapy reported to the app and questionnaire (Days 1 to 7).

Description of participants	App				Questionnaire		
	Participants, n	Coefficient^a^	*P* value^b^	Participants, n		Coefficient^a^	*P* value^b^
Reported smoking abstinence	5	0.205	.74	4		–0.316	.68
All^c^	14	0.184	.55	13		0.031	.92

^a^Spearman rank-order correlation coefficients are presented unless otherwise stated.

^b^*P* value is two-tailed.

^c^Partial Spearman correlation coefficient adjusting for mean daily number of cigarettes reported to either the app or questionnaire, as appropriate; one participant in these analyses reported using an electronic cigarette, but removing them had little effect on *P* values.

**Table 3:** Days 1 to 7 smoking and nicotine replacement therapy (NRT) status reported to the app and questionnaire.

Behavior reported	App (n=31), n (%)	Questionnaire (n=26), n (%)
Abstinent (did not smoke “even a puff”)	9 (29)	6 (23)
Used an electronic cigarette	3 (10)	2 (8)
Used any study NRT	31 (100)	26 (100)
Used any nonstudy NRT	1 (3)	0 (0)

## Discussion

### Principal Findings

In pregnant women who were trying to stop smoking, we measured adherence to NRT for 28 days with a daily smartphone app survey and retrospective questionnaires sent at 7 and 28 days following a QD. With an identical maximum value of compensation for participants’ time taken to complete measures (£25 per method), more participants provided some app data than completed either of the questionnaires, and compliance with daily app surveys was encouraging, with respondents completing an average of 25 out of 28 reports. Women reported using NRT on more days and in higher daily doses to questionnaires, and this may have been due to overreporting of NRT use to questionnaires.

### Limitations

As participants were self-selected, pregnant, recruited in a health care setting, and agreed to try using NRT to stop smoking and to receive a bespoke intervention to enhance their NRT use, the generalizability of findings, especially to nonpregnant populations, could be questioned. Participants will probably have been more interested in and motivated to use NRT than other pregnant women, and so may have been more strongly orientated toward recording NRT use as requested. However, one would expect this to increase completion rates for both reporting methods; it is less plausible as an explanation for differences.

The study could be criticized for not randomizing participants to data collection methods. We did not have the resources for a randomized controlled trial solely to determine the best way to collect NRT adherence data. Therefore, we instead interpret differences between within-subject data collected by two methods for a pragmatic study set in a routine clinical setting. The within-subjects design, with questionnaire data collection following app data collection, may mean that study fatigue caused lower questionnaire response rates; however, response rates were similar for Day 7 and Day 28 questionnaires, so this may not have been an issue.

It is possible that different payment schedules affected the relative response rates for questionnaires versus the app. Although the maximum compensation available for data completion was equal and small for each method (£25), questionnaire completion attracted a one-off payment, whereas app completion attracted a small daily amount (£0.50) plus bonuses for complete weeks, with the total to date displayed on the app interface; this could obviously encourage app usage. However, it could be argued that the app required more of participants’ effort for the same overall compensation, and that those failing to report on the app every day might become demotivated. Moreover, payments (shopping vouchers) for both methods were not made until the study ended for a participant. We therefore consider it unlikely that this can explain the greater response rate for the app, although it is likely that responses for both methods would have been lower given no compensation for participants’ time.

The study could also be criticized for not validating reported NRT use other than via exploratory comparison with salivary cotinine concentrations. Participants were not asked to return nonused NRT or empty NRT packaging as evidence of having used NRT. Although such measures have occasionally been used to validate reports of adherence to smoking cessation treatments [20], there is no guarantee that participants would actually have used nonreturned NRT, and there is currently no “gold-standard” means for measuring adherence. As compensation for completion of measures was offered regardless of responses given (eg, NRT used or not), there was no financial motivation for participants to deliberately misreport using NRT.

The principal finding that, compared to app reporting, questionnaire data may overreport adherence is based on the repeated observation of instances in which participants specifically reported *not* using NRT to the app on one or more days, but later reported to questionnaires that NRT *was* used on these days. Additionally, some participants appeared to report very high mean daily nicotine doses to the questionnaire but not to the app. It is plausible that app data may have underestimated NRT use, as we assumed that no NRT was used on days when no app report was made; however, we found clear examples where participants reported greater NRT use to questionnaires when app data were present for the same period. It is possible that, relative to the app, social desirability may have affected questionnaire data collection carried out by telephone (ie, in Cohorts 1 and 2), although when questionnaire data were collected by online survey in Cohort 3, the tendency to report having used NRT on all 28 days was still high. Hence, we think it probable that app data reported a maximum of 48 hours after NRT use were more accurate than questionnaire data recalled 7 or 28 days later, and the skew toward questionnaire reports of NRT use on all days in a recall period reflects this effect. Given the study design, there may be alternative explanations; however, as recall of events is probably better closer to their occurrence [27], we think that this is the most plausible explanation.

The exploratory nature of comparisons between average daily nicotine dose and Day 7 cotinine levels should be emphasized. Cotinine has a 19-hour half-life and can remain in saliva for up to 1 week after nicotine exposure; hence, comparing mean daily nicotine doses over the previous 7 days with saliva cotinine concentrations at Day 7 is logical. However, due to accelerated nicotine metabolism in pregnancy, the cotinine half-life of women in this study could be as short as ~9 hours [14], and we cannot be sure how this might have affected the findings. The sample size for this analysis was too small to permit adjusting for potential confounders of cotinine concentrations, such as partner smoking status or nicotine metabolic rate; thus, the findings from these analyses need to be treated with caution. Numbers of participants not smoking “even a puff” between QD and Day 7 were very low, and therefore our correlations based on more than 10 participants relied on adjusting for the numbers of daily cigarettes smoked, which was self-reported and therefore potentially prone to error. In this study, however, we found collection of data on cotinine concentrations to be feasible and, given larger sample sizes, this could be a useful method for validating self-report data on nicotine use in future studies.

### Strengths

Study originality is a strength; few studies have addressed how to best measure adherence to smoking cessation medication, and no such study has been conducted within pregnant women to date [20]. We believe this is the first comparison of methods for NRT adherence measurement, and the first to investigate the relationships between self-report measures of daily NRT dose used and saliva cotinine concentrations. Although in the latter analyses correlation coefficients were imprecise, these are the first and are therefore the best coefficient estimates available. The hard-to-reach nature of the study population (people who smoke in pregnancy) is another study strength as remote data collection methods are ideally suited to such populations, suggesting ecological validity. Additionally, it is plausible that the study results and data collection methods could be generalized/adapted to other medications; medication adherence remains a vital area of health research.

### Implications

Daily reporting of NRT adherence via an app may have several potential advantages over retrospective reporting at study follow-ups. First, the accuracy of participant recall is likely to be better with shorter intervals since a behavior occurred; this is central to the EMA approach to data collection, which aims to minimize recall bias and maximize ecological validity [27]. Without keeping records, participants may struggle to accurately recall the number of days on which they used NRT given longer time intervals. This seems to have occurred in this study as, for some participants, higher retrospectively reported NRT usage was explicitly contradicted by daily app reports of days when no NRT was used, particularly at Day 28. It is also possible that, with retrospective measures, participants are unclear about the exact period they are supposed to be reporting for, particularly if there is a delay in completion of measures. The precise amounts of fast-acting NRT used are likely to be even more difficult to recall or prone to error. Several large outliers were evident for the Day 7 questionnaire measure of NRT dosage relative to the app, and it is possible that these participants reported a weekly total rather than daily average. Consequently, daily adherence reporting may be preferential for measuring adherence to NRT in trials, which often have treatment periods of 4 weeks or more and where fast-acting NRT might be used, and an app provides an ideal tool for collecting daily measurements.

A second potential advantage is that app data are available to researchers as soon as they are submitted. This means that, with daily app reports, participants lost to follow-up may still provide useful adherence data right up until the point of dropout or withdrawal, whereas periodic or end-of-study measures can only be completed by participants retained in studies. In this study, more than half of participants with no questionnaire data had provided some app data, particularly within the first 7 days. This could permit better estimates of treatment adherence in trials and provide informative data on those who withdraw.

One potential problem with daily adherence reporting is that monitoring one’s own treatment adherence (“self-monitoring”) could make adherence more likely; that is, the act of measurement may affect the behavior [20,27]. However, in a research setting, this can likely be minimized by storing no usage data on the app, so that users cannot review their adherence with treatment, and by configuring apps to provide no other feedback on adherence to users. Enabling self-monitoring of NRT use via the app may be a desirable feature to incorporate in a clinical setting. Another important criterion to consider when judging the utility of a reporting measure, as well as data accuracy and completeness, is participant burden. EMA data such as daily app reports are likely to be more accurate but may also be more burdensome for participants than less frequent questionnaires, and a balance must be made between these needs. Less frequent questionnaires may be adequate in studies if they are found to replicate EMA data closely, and weekly questionnaires are likely to be more valid than monthly questionnaires.

### Conclusions

Using a smartphone app to request daily information on NRT use may provide more complete and valid data on adherence to NRT than using periodically administered questionnaires with longer recall periods. Although this analysis was conducted within a small sample of pregnant women, it seems likely that NRT usage reporting patterns will be similar for nonpregnant people, and apps could provide better data than questionnaires for other drug treatments.

## Appendix

Multimedia Appendix 1 Study dates and details of questionnaire data collection per cohort (Table S1); app and questionnaire items per cohort; participant-level NRT adherence data reported to app and questionnaire for Days 1-7 (Table S2); participant-level NRT adherence data reported to app and questionnaire for Days 1-28 (Table S3); and partial regression plots between self-reported mean daily nicotine dose from NRT and Day 7 cotinine concentration, for app and questionnaire, adjusted for self-reported daily number of cigarettes (Figure S1).

## Abbreviations

BMN: Baby, Me & NRT
e-Cigarette: electronic cigarette
EMA: ecological momentary assessment
NIHR: National Institute for Health Research
NRT: nicotine replacement therapy
QD: quit date

## Data Availability

The data underlying this article will be shared on reasonable request to the corresponding author.
