# Supplementary material for: Comparison of a Daily Smartphone App and Retrospective Questionnaire Measures of Adherence to Nicotine Replacement Therapy Among Pregnant Women: Observational Study
Source: JMIR Form Res. 2023 Mar 7;7:e35045. doi: 10.2196/35045 (PMC10031440; doi:10.2196/35045)
Supplement: Multimedia Appendix 1 [file formative_v7i1e35045_app1.docx]

**Multimedia Appendix 1: Supplementary Information**

**Table S1. Study dates and details of questionnaire data collection per cohort**

| **Cohort details** | **Timing of questionnaire data collection** | **How questionnaire data were obtained** | **Summary of questionnaire NRT adherence measures** |
| --- | --- | --- | --- |
| **Cohort 1:**  8 participants  Recruited between 09.10.19 & 13.11.19 | 7, 14, 21 & 28 days after quit date (QD) | **All time points**:  Interview (face-to-face or telephone) with researcher who delivered smoking cessation intervention. Participants had the choice of a face-to-face or telephone interview. All Day 7 and Day 28 interviews were carried out face-to-face. | **Recall period**  ***Days 7, 14, 21, 28:***7-day recall period, participants were asked ‘*In the last week, have you used any NRT?’*    **Quantification**  ***Patches:*** If patch use reported, participants were asked ‘*How many have you used*?’ over the recall period  ***Fast-acting NRT:*** If fast-acting NRT use reported, participants were asked ‘O*n average, how many lozenges/cartridges have you used each day?*’ over the recall period |
| **Cohort 2:**  12 participants  Recruited between 03.02.20 & 13.03.20 | 7 & 28 days after QD | **Both time points**: Interview (face-to-face or telephone) with researcher who delivered smoking cessation intervention. Participants had the choice of a face-to-face or telephone interview until Covid-19 restrictions meant that only the telephone method was possible. 3/10 Day 7 and 8/9 Day 28 interviews were carried out by telephone. | **Recall period**  ***Day 7*:**7-day recall period, participants were asked *‘In the 7 days since your QD, on how many days have you used any NRT?’*  ***Day 28***: 28-day recall period, participants were asked ‘*In the 28 days since your QD, on how many days have you used any NRT?’*    **Quantification**  ***Patches:*** Minor changes, wording essentially as Cohort 1, participants were asked ‘*How many of the NRT patches have you used*?’ over the recall period  ***Fast-acting NRT:*** Participants were asked ‘*on how many days*’ fast-acting NRT was used over the recall period and, only for days when used, ‘*how many lozenges/cartridges have you used on average each day?’* |
| **Cohort 3:**  20 participants  Recruited between 20.08.20 & 30.09.20 | 7 & 28 days after QD | **Both time points**: Online questionnaire with link sent via text message and email, or via telephone if no response, by a researcher who did not deliver the smoking cessation intervention. | **Recall period**  ***Day 7****:*  7-day recall period, wording as Cohort 2  ***Day 28****:* 28-day recall period, wording as Cohort 2    **Quantification**  ***Patches:*** Participants were asked, ‘*On how many days have you used NRT patches?’* over the recall period  ***Fast-acting NRT:*** Wording as Cohort 2 |

**App items per cohort**

**Cohort 1, all 28 days**

1. Did you smoke tobacco on (date), even just a puff? (do not count e-cigarettes)
   1. *(y/n)*
2. Did you use NRT on (date)? (do not count e-cigarettes)
   1. *(y/n)*
3. *(if yes)* Which types of NRT did you use on (date)?
   1. Patches *(tickbox and number)*

*(if ticked)* Please count only new patches applied on that date – do not count patches put on the day before e.g. left on overnight

- 1. Inhalator cartridges *(tickbox and number)*

*(if ticked)* Please count how many inhalator cartridges you used or partially used on that date – count 1 cartridge if you used less than a whole one, 2 cartridges if you used 1 to 2, and so on

- 1. Lozenges *(tickbox and number)*

*(if ticked)* Please count how many lozenges / mini lozenges you used on that date

1. Did you use any other type of NRT on (date) which was not given to you by the study team? (do not include e-cigarettes)
   1. *y/n*
2. Did you use an e-cigarette on (date)?
   1. *y/n*

**Cohorts 2 and 3, all 28 days**

Items as above but with the following additions to items 1 and 3:

1. Did you smoke tobacco on (date), even just a puff? (do not count e-cigarettes)
   1. *(if yes) Please enter how many cigarettes you smoked (number)*
2. Which types of NRT did you use on (date)?
   1. Mouth spray *(tickbox)*

*(if ticked)* Please count how many times you used the spray on that date *(number)*

**Questionnaire items per cohort**

**Cohort 1, Day 7**

- (If currently smoking) How many cigarettes per day are you smoking currently?
- In the last week, have you used an e-cigarette?
- In the last week, have you used any of the NRT that the study team provided?
- In the last week, have you used any of the NRT patches that the study team provided?
  - If 'Yes': How many have you used?
- In the last week, have you used the NRT inhalator that the study team provided?
  - If ‘Yes’: On average, how many inhalator cartridges have you used each day?
- In the last week, have you used the NRT lozenges that the study team provided?
  - If ‘Yes’: On average, how many lozenges have you used each day?

**Cohort 1, Day 28**

Items as above

**Cohort 2, Day 7**

- (If currently smoking) How many cigarettes per day are you smoking currently?
- In the 7 days since your quit date, have you used an e-cigarette?
- In the 7 days since your quit date, on how many days have you used any NRT?
- In the 7 days since your quit date, how many of the NRT patches have you used?
- In the 7 days since your quit date, on how many days have you used the NRT inhalator?
  - (if used) Over those days, how many inhalator cartridges on average have you used each day?
- In the 7 days since your quit date, on how many days have you used the NRT lozenges?
  - (if used) Over those days, how many lozenges on average have you used each day?

**Cohort 2, Day 28**

Items as above but asking “In the 28 days since your quit date ….”

**Cohort 3, Day 7**

Items as above but with the following amendment and addition:

- In the 7 days since your quit date, on how many days have you used NRT patches?
- In the 7 days since your quit date, on how many days have you used the NRT Quick Mist?
  - (if used) Over those days, how many sprays of the Quick Mist on average have you used each day?

**Cohort 3, Day 28**

Items as above but asking “In the 28 days since your quit date ….”

**Table S2: Participant-level NRT adherence data reported to app and questionnaire for Days 1-7**

|  | **Number of days of any NRT use reported for Days 1 to 7 recall period** | | | | |
| --- | --- | --- | --- | --- | --- |
|  | **Reported to questionnaire** | **Reported to app** | | | |
| **Participant ID** | **Days any NRT used ^a^**  (‘-’ no questionnaire data) | **Days any NRT used** | **Days no NRT used** | **Days no app data** | **Total days reported** |
| **Cohort 1** | | | | | |
| 1101 | - | 2 | 1 | 4 | 3 |
| 1102 | 7 | 7 | 0 | 0 | 7 |
| 1103 | 7 | 5 | 1 | 1 | 6 |
| 2101 | 3^b^ | 4 | 1 | 2 | 5 |
| 2102 | 7 | 7 | 0 | 0 | 7 |
| 2103 | 0^b^ | 2 | 0 | 5 | 2 |
| 2104 | - | 0 | 0 | 7 | 0 |
| 2105 | 10^c^ | 6 | 1 | 0 | 7 |
| **Cohort 2** | | | | | |
| 1201 | - | 3 | 0 | 4 | 3 |
| 1202 | 7 | 7 | 0 | 0 | 7 |
| 1203 | 7 | 6 | 1 | 0 | 7 |
| 1204 | 3 | 2 | 5 | 0 | 7 |
| 1205 | 7 | 6 | 1 | 0 | 7 |
| 2201 | 5 | 4 | 3 | 0 | 7 |
| 2202 | 7 | 7 | 0 | 0 | 7 |
| 2203 | 7 | 7 | 0 | 0 | 7 |
| 2204 | 7 | 7 | 0 | 0 | 7 |
| 2205 | 3 | 2 | 1 | 4 | 3 |
| 2206 | 7 | 6 | 0 | 1 | 6 |
| 2207 | - | 2 | 1 | 4 | 3 |
| **Cohort 3** | | | | | |
| 1301 | 8 | 6 | 0 | 1 | 6 |
| 1302 | 7 | 7 | 0 | 0 | 7 |
| 1303 | - | 0 | 0 | 7 | 0 |
| 1305 | 7 | 7 | 0 | 0 | 7 |
| 1306 | 7 | 7 | 0 | 0 | 7 |
| 1307 | - | 0 | 0 | 7 | 0 |
| 2301 | - | 0 | 0 | 7 | 0 |
| 2303 | 7 | 7 | 0 | 0 | 7 |
| 2304 | - | 2 | 0 | 5 | 2 |
| 2306 | 7 | 7 | 0 | 0 | 7 |
| 3301 | 7 | 7 | 0 | 0 | 7 |
| 3302 | 9 | 7 | 0 | 0 | 7 |
| 3303 | 4 | 4 | 3 | 0 | 7 |
| 3305 | - | 5 | 0 | 2 | 5 |
| 3306 | 7 | 7 | 0 | 0 | 7 |

^a^ For Cohort 1, this is based on the ‘number of patches’ used in any recall period, assuming that one patch was used per day. For Cohorts 2 and 3 we asked for the ‘number of days’ on which any NRT was used.

^b^ This figure, based solely on the ‘number of patches’ for Cohort 1, may be an underestimate of the number of days of NRT use for this participant. The participant reported using some fast-acting NRT, but the number of days was unknown.

^c^ Participant reported using more than seven patches in one week; we assume that patches were used on all seven days.

**Table S3: Participant-level NRT adherence data reported to app and questionnaire for Days 1-28**

|  | **Number of days of any NRT use reported for Days 1 to 28 recall period** | | | | |
| --- | --- | --- | --- | --- | --- |
|  | **Reported to questionnaire** | **Reported to app** | | | |
| **Participant ID** | **Days any NRT used ^a^**  (‘-’ no questionnaire data) | **Days any NRT used** | **Days no NRT used** | **Days no app data** | **Total days reported** |
| **Cohort 1** | | | | | |
| 1101 | - | 2 | 1 | 25 | 3 |
| 1102 | 24 | 23 | 1 | 4 | 24 |
| 1103 | 28 | 24 | 1 | 3 | 25 |
| 2101 | 4^b^ | 9 | 7 | 12 | 16 |
| 2102 | 30^c^ | 27 | 0 | 1 | 27 |
| 2103 | 0^b^ | 4 | 0 | 24 | 4 |
| 2104 | - | 0 | 0 | 28 | 0 |
| 2105 | 18^c^ | 15 | 13 | 0 | 28 |
| **Cohort 2** | | | | | |
| 1201 | - | 3 | 0 | 25 | 3 |
| 1202 | 28 | 25 | 0 | 3 | 25 |
| 1203 | 28 | 26 | 2 | 0 | 28 |
| 1204 | 28 | 21 | 5 | 2 | 26 |
| 1205 | 28 | 12 | 10 | 6 | 22 |
| 2201 | 10 | 4 | 23 | 1 | 27 |
| 2202 | 28 | 22 | 0 | 6 | 22 |
| 2203 | 28 | 23 | 0 | 5 | 23 |
| 2204 | 28 | 28 | 0 | 0 | 28 |
| 2205 | 15 | 7 | 15 | 6 | 22 |
| 2206 | - | 6 | 0 | 22 | 6 |
| 2207 | - | 2 | 3 | 23 | 5 |
| **Cohort 3** | | | | | |
| 1301 | 24 | 22 | 5 | 1 | 27 |
| 1302 | - | 7 | 0 | 21 | 7 |
| 1303 | - | 0 | 0 | 28 | 0 |
| 1305 | 28 | 25 | 0 | 3 | 25 |
| 1306 | 28 | 28 | 0 | 0 | 28 |
| 1307 | - | 0 | 0 | 28 | 0 |
| 2301 | - | 0 | 0 | 28 | 0 |
| 2303 | 28 | 28 | 0 | 0 | 28 |
| 2304 | - | 6 | 0 | 22 | 6 |
| 2306 | 28 | 24 | 1 | 3 | 25 |
| 3301 | 23 | 25 | 2 | 1 | 27 |
| 3302 | 28 | 28 | 0 | 0 | 28 |
| 3303 | - | 15 | 4 | 9 | 19 |
| 3305 | 28 | 25 | 0 | 3 | 25 |
| 3306 | 28 | 28 | 0 | 0 | 28 |

^a^ For Cohort 1, this is based on the ‘number of patches’ used in any recall period, assuming that one patch was used per day. For Cohorts 2 and 3 we asked for the ‘number of days’ on which any NRT was used. For Cohort 1, NRT use over the 28-day period was calculated by summing responses to the four weekly questionnaires.

^b^ This figure, based solely on the ‘number of patches’ for Cohort 1, may be an underestimate of the number of days of NRT use for this participant. The participant reported using some fast-acting NRT, but the number of days was unknown.

^c^ Participant reported using more than seven patches in one week.

**
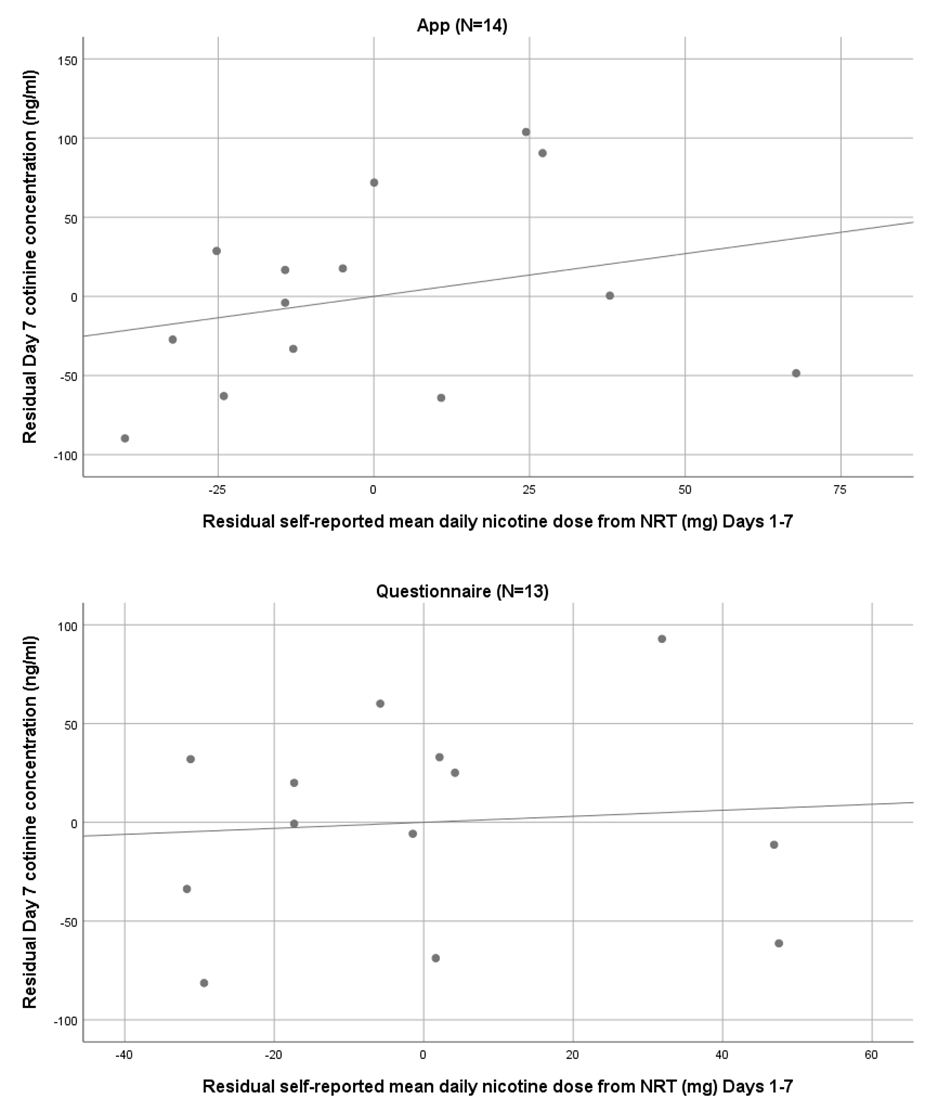
**

**Figure S1: Partial regression plots between self-reported mean daily nicotine dose from NRT and Day 7 cotinine concentration, for app and questionnaire, adjusted for self-reported daily number of cigarettes (see Table 2, row ii)**
